# Establishing a Multicolor Flow Cytometry to Characterize Cellular Immune Response in Chickens Following H7N9 Avian Influenza Virus Infection

**DOI:** 10.3390/v12121396

**Published:** 2020-12-06

**Authors:** Xiaoli Hao, Shuai Li, Lina Chen, Maoli Dong, Jiongjiong Wang, Jiao Hu, Min Gu, Xiaoquan Wang, Shunlin Hu, Daxin Peng, Xiufan Liu, Shaobin Shang

**Affiliations:** 1 College of Veterinary Medicine, Yangzhou University, Yangzhou 225009, China; xlhao@yzu.edu.cn (X.H.); lsmuzi98@163.com (S.L.); CLN18762314118@163.com (L.C.); 18627057287@163.com (M.D.); wjiongjiong@163.com (J.W.); hujiao@yzu.edu.cn (J.H.); gumin@yzu.edu.cn (M.G.); wxq@yzu.edu.cn (X.W.); slhu@yzu.edu.cn (S.H.); pengdx@yzu.edu.cn (D.P.); 2 Institute of Comparative Medicine, Yangzhou University, Yangzhou 225009, China; 3 Jiangsu Co-innovation Center for Prevention and Control of Important Animal Infectious Diseases and Zoonosis, Yangzhou University, Yangzhou 225009, China; 4 International Corporation Laboratory of Agriculture and Agricultural Products Safety, Yangzhou University, Yangzhou 225009, China

**Keywords:** cellular immune response, flow cytometry, avian influenza virus, H7N9, chicken

## Abstract

Avian influenza virus (AIV) emerged and has continued to re-emerge, continuously posing great threats to animal and human health. The detection of hemagglutination inhibition (HI) or virus neutralization antibodies (NA) is essential for assessing immune protection against AIV. However, the HI/NA-independent immune protection is constantly observed in vaccines’ development against H7N9 subtype AIV and other subtypes in chickens and mammals, necessitating the analysis of the cellular immune response. Here, we established a multi-parameter flow cytometry to examine the innate and adaptive cellular immune responses in chickens after intranasal infection with low pathogenicity H7N9 AIV. This assay allowed us to comprehensively define chicken macrophages, dendritic cells, and their MHC-II expression, NK cells, γδ T cells, B cells, and distinct T cell subsets in steady state and during infection. We found that NK cells and KUL01^+^ cells significantly increased after H7N9 infection, especially in the lung, and the KUL01^+^ cells upregulated MHC-II and CD11c expression. Additionally, the percentages and numbers of γδ T cells and CD8 T cells significantly increased and exhibited an activated phenotype with significant upregulation of CD25 expression in the lung but not in the spleen and blood. Furthermore, B cells showed increased in the lung but decreased in the blood and spleen in terms of the percentages or/and numbers, suggesting these cells may be recruited from the periphery after H7N9 infection. Our study firstly disclosed that H7N9 infection induced local and systemic cellular immune responses in chickens, the natural host of AIV, and that the flow cytometric assay developed in this study is useful for analyzing the cellular immune responses to AIVs and other avian infectious diseases and defining the correlates of immune protection.

## 1. Introduction

Avian influenza A (H7N9) virus has posed a dual challenge to public health and the poultry industry since its emergence in 2013 in China [1]. Although the isolation of H7N9 AIV field strains have dropped dramatically in both chicken and humans after massive immunization of poultry with H5/H7 vaccine in chickens since 2017 in China [2], H7N9 variants that are well-adapted in waterfowls have surfaced recently in poultry with higher pathogenicity in chickens and have caused human infection [2,3]. Many vaccine candidates against AIV H7N9 subtype, including inactivated vaccine [4] and HA-carrying recombinant vector vaccine [5,6] have been developed and experimentally shown protection against H7N9 in mice and humans [7]. However, these vaccines induced a low level of hemagglutination inhibition (HI), and viral neutralizing antibodies (NA) titers against the H7N9 subtype that was lower than the standard of vaccine evaluation (>2^4^) for other influenza A subtypes (e.g., H1N1 and H5N1) [8] and seasonal influenza viruses. They were also not well correlated with the immune protection conferred by these vaccines [5,6]. Similarly, in chickens, our previous studies showed that recombinant Newcastle disease virus (NDV) vaccine carrying H7N9 HA gene (rNDV-H7N9 HA) provided complete protection but did not induce a high level of HI and NA [9,10]. This suggests that HI/NA-independent immune protection may play dominant roles, especially for the H7N9 subtype, and that the detection of a humoral response by traditional serological assay may not be sufficient to evaluate the efficacy of vaccines [11] properly. Thus, it is necessary to develop new methods to analyze cellular immune response in poultry after infection or vaccination [12].

In addition to the humoral response, it is well established that cell-mediated immunity plays an important role in protecting against influenza A virus (IAV) in mammals [13,14,15]. IAV infects airway epithelial cells and triggers the expression of inflammatory cytokines and chemokines, which recruit innate immune cells such as neutrophils, monocytes, and macrophages into the lung to dampen the early stages of IAV infection [16] or promote the immunopathology. In the meantime, respiratory dendritic cells (DCs) become maturated, upregulating the expression of major histocompatibility complex class II (MHC-II) and co-stimulatory molecules, sampling and processing viral antigens to present to CD4 and CD8 T cells [17,18] and initiating the priming of adaptive immune responses. Both CD4 and CD8 T cells can confer homologous and heterologous protection against influenza virus in an antigen-specific manner [13,15]. In addition, NK cells are required for the clearance of influenza virus [19,20], and γδ T cells are activated and show a protective function to human and avian (H5N1) influenza virus [14,21,22]. While the cellular immunity against influenza A virus in mice and humans has been extensively investigated [13,14], the analysis of cellular immune response in poultry after AIV infection or vaccination has long been neglected.

Chickens are natural hosts for AIV and responsible for the emergence of novel AIV subtypes and their transmission to humans [2]. To better understand the immune response of chicken after AIV infections would facilitate the development of new control strategies in poultry. By depleting chicken CD8 T cells, it has been demonstrated that chicken CD8 T cells can mediate protection against the H5N1 subtype [23]. CD3^−^CD8α^+^ NK cells in the lung were found to be activated differentially after AIV H5N1 and H9N2 infection and correlated inversely with pathogenicity in chicken [24]. How chicken immune cells other than CD8 and NK cells respond to AIV infection or immunization has not been examined.

Flow cytometry has emerged as an essential and powerful tool that offers to simultaneously detect multiple parameters at a single cell level and precisely define the phenotypes and functions of distinct immune cell subsets [25]. In recent years, flow cytometry was applied in chicken to differentiate leukocytes from thrombocytes by detecting CD45 expression and side scatter properties [26] to assess the frequency of chicken CD4, CD8, and γδ T cells and their expression of activation markers CD44 and CD45 after NDV infection [27,28], and to quantify absolute numbers of white blood cells in blood including thrombocyte, monocyte, T-cell, B-cell, and heterophilic granulocyte, without the need to remove nucleated erythrocytes and thrombocytes [29]. However, flow cytometric assessment of the cellular immune response of chicken after AIV infection, in particular H7N9 subtype, has not been investigated.

In this study, we developed a multi-parameter flow cytometry by using a combination of antibodies to chicken markers Bu-1, monocyte/macrophage (KUL01), CD45, MHC-II, CD11c, CD3, CD8α, CD8β, TCRγδ, CD4, and CD25 to comprehensively numerate innate and adaptive immune cell populations in chicken at steady state and after H7N9 infection, leading to an in-depth analysis of the cellular composition and dynamics within lymphoid and non-lymphoid tissues. We found H7N9 infection induced distinct local and systemic cellular immune responses in chickens.

## 2. Materials and Methods

### 2.1. Ethics Statement

All experiments involving live H7N9 viruses were approved by the Institutional Biosafety Committee of Yangzhou University and were performed in animal biosafety level 3 (ABSL-3) facilities in accordance with the institutional biosafety manual (CNAS BL0015). The protocols for all animal studies were approved by Jiangsu Province Administrative Committee for Laboratory Animals (approval number: SYXK-SU-2017-0007) and complied with the guidelines of Jiangsu Province Laboratory Animal Welfare and ethics of Jiangsu Province Administrative Committee of Laboratory Animals.

### 2.2. Virus and Animal Experiments

A low pathogenic AIV (LPAIV) H7N9 (A/Chicken/Anhui/AH395/2017) was used for the infection of chickens [30]. Viruses were propagated in specific pathogen-free (SPF) embryonated chicken eggs. Four-week-old SPF White Leghorn chickens were divided into two groups. One group was mock-infected (uninfected) and the other group was infected intranasally (i.n.) with 10^5.0^ EID_50_ of the virus in 0.1 mL (infected). Six chickens per group were humanely euthanized at 7, 14, and 28 days post-infection (dpi), and tissue samples including spleen, cecal tonsil, lung, thymus, bursa, blood, and bone marrow were harvested for cell isolation.

### 2.3. Cell Isolation

Single-cell suspensions from tissues were prepared as in a previous study [31], and peripheral blood mononuclear cells (PBMC) were isolated from blood as per previous reports [32]. Briefly, the organs were mechanically disrupted and pushed through a 70-μm cell strainer (Corning Inc., Corning, NY, USA) using the plungers of 5 mL syringe. The cells were resuspended in 10 mL with phosphate-buffered saline (PBS) containing 2% fetal bovine serum (FBS). Lung was cut into small pieces and digested with collagenase IV (1 mg/mL; Sigma, St. Louis, MO, USA) and DNase I (30 µg/mL; Sigma, St. Louis, MO, USA) for 30 min at 37 °C before disruption. Cell suspensions were overlaid onto the tissue separation medium at a 1:1 ratio to isolate mononuclear cells according to the manufacturer’s instructions (Haoyang, Tianjin, China). After centrifugation at room temperature for 30 min at 500× *g*, the interface was collected and washed twice with PBS. After centrifugation at 400× *g* for 10 min, cells were resuspended in 5 mL of complete medium (CM; RPMI-1640 supplement with 10% FBS (Gibco, Grand Island, NY, USA), 1% penicillin plus streptomycin (Invitrogen, Carlsbad, CA, USA)). To isolate PBMCs, whole blood containing anti-coagulant heparin sodium was diluted with an equal volume of PBS and layered on Histopaque-1077 (Sigma-Aldrich, Poole, UK) and subjected to the above procedures. Red blood cells were lysed with RBC lysis buffer (Gibco, Grand Island, NY, USA) for 5 min. Bone marrow cells were isolated, as described previously [33]. Briefly, bone marrow cells were flushed out from bones with PBS and pushed through a 70 μm nylon cell strainer, and the resultant cell suspensions were loaded onto an equal volume of Histopaque-1119 (Sigma-Aldrich, Poole, UK) and centrifuged at 1200× *g* for 30 min. Cells at the interface were collected, washed twice with PBS, and resuspended in CM. Cells were counted using a hemocytometer (Sigma-Aldrich, St. Louis, MO, USA) and trypan blue, and the final cell concentration was adjusted to 2 × 10^7^ live cells/mL.

### 2.4. Flow Cytometry

Cells were plated on 96-well V-bottom plate with 2 × 10^6^ cells each well in 100 μL FACS buffer (0.5% FBS in PBS). Monoclonal antibodies (mAb) specific for chicken B cells (Bu-1), monocyte/macrophage (KUL01), CD45, MHC-II, CD3, CD8α, CD8β, TCRγδ, CD4, and CD25 with different fluorochrome conjugate were purchased from Southern Biotech (Birmingham, AL, USA) and polyclonal antibody (pAb) to human CD11c were purchased from Beijing Biosynthesis Biotechnology Co., Ltd. (Bioss, Beijing, China) (Table 1). Two panels of antibody cocktails were made to distinguish different immune cells: Panel 1 was used to identify chicken B-cells and myeloid lineage (monocytes, macrophages, and dendritic cells) containing anti-monocyte/macrophage-PE, anti-CD45-PerCP-Cy5.5, anti-Bu-1-FITC, anti-MHC-II-PE/cy7, and anti-CD11c-APC. Panel 2 was used to define chicken T cell subsets, NK cells and their activation state containing anti-TCRγδ-BV510, anti-CD3-PerCP-Cy5.5, anti-CD4-Pacific blue, anti-CD8β-PE, anti-CD8α-Alexa eFluor 700, and anti-CD25-FITC. Isotype antibodies or fluorescence minus one (FMO) for CD11c, MHC-II, and CD25 were used to set gating. The cells were incubated with 1% chicken serum to block FC receptors and then stained with fixable viability dye (FVD) eFluor 780 (Thermo Fisher Scientific, Waltham, MA, USA) for excluding dead cells. After centrifugation, a final volume of 50 μL antibody cocktail was added to the cells and incubated for 30 min at 4 °C. After washing twice and centrifugation at 400× *g* for 5 min at 4 °C, the cells were resuspended in 200 μL PBS for FACS analysis. Flow cytometry was performed with a FACS LSRFortessa (BD Biosciences, Franklin Lakes, NJ, USA) and a minimal number of 100,000 cells was acquired. Data analysis was processed by FlowJo software (Tree Star Inc., Ashland, OR, USA), and the total cell number of indicated subset per organ or 5 mL blood was subsequently calculated.

### 2.5. Statistical Analysis

Statistically significance between experimental groups was determined using the independent-samples t-test in the SPSS statistics software (SPSS 23.0, IBM, Ehningen, Germany). *p* values < 0.05 were defined as statistically significant.

## 3. Results

### 3.1. Establishing a Polychromatic Flow Cytometry to Define Different Immune Cells in Chicken

**Figure 1:**
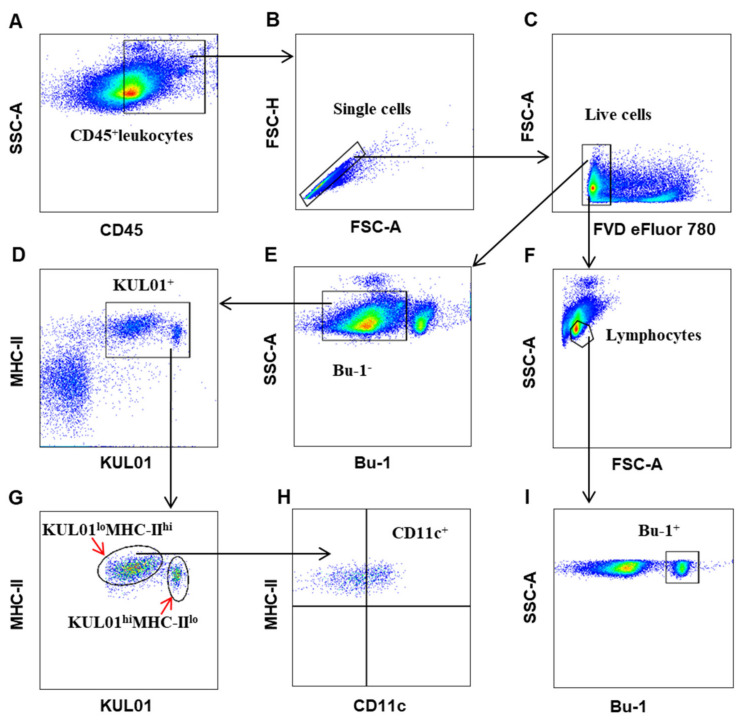
Gating strategies to identify chicken B-cells and myeloid lineage. Mononuclear cells were isolated from spleens of 4 weeks-old naïve chicken and surface stained with antibody cocktails (panel 1: CD45, Bu-1, KUL01, MHC-II, CD11c, and FVD eFluor 780). The leukocytes were gated with CD45 positive (**A**) and then single cells were gated based on size and granularity using FSC-A and FSC-H (**B**). Live cells were defined as FVD eFluor 780-negative (**C**). Then, the lymphocytes were chosen (**F**) using forward scatter (FSC)-A and SSC-A, and Bu-1^+^ B cells were defined (**I**). By excluding Bu-1^+^B cell (**E**) and outputting KUL01 vs MHC-II, the KUL01^+^ cells were identified (**D**), which were further subdivided into KUL01^lo^MHC-II^hi^ and KUL01^hi^MHC-II^lo^ cells (**G**). Finally, partial CD11c expression was confirmed on the KUL01^lo^MHC-II^hi^ cells but not on the KUL01^hi^MHC-II^lo^ cells (**H**).

**Figure 2:**
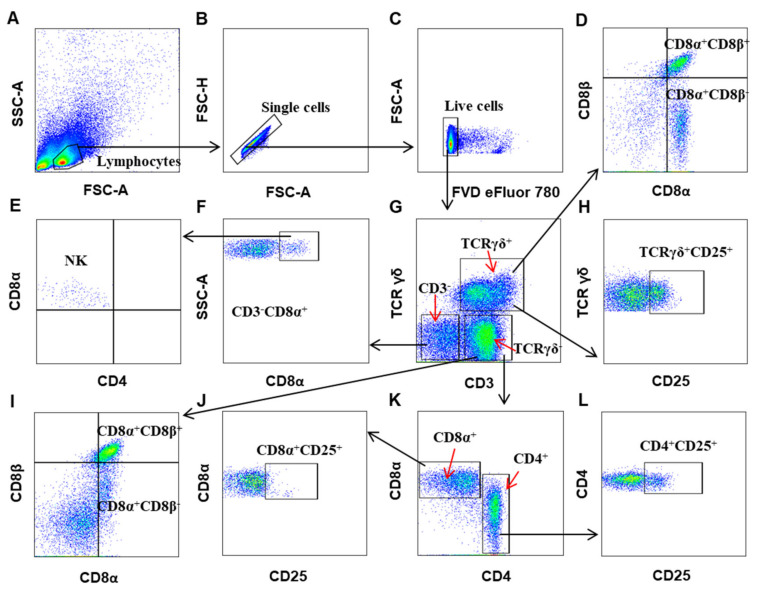
Gating strategies to define chicken T cell subsets, NK cells, and their activation state. Mononuclear cells were isolated from spleens of 4 weeks-old naïve chicken and surface stained with antibody cocktails (Panel 2: CD3, TCRγδ, CD4, CD8α, CD8β, CD25, and FVD eFluor 780). The lymphocytes are chosen with FSC versus SSC (**A**), and then single cells were gated using FSC-A and FSC-H (**B**). Live cells were defined as FVD eFluor 780-negative (**C**). By outputting CD3 versus TCRγδ, CD3^+^TCRγδ^+^ (γδ T cells), CD3^+^TCRγδ^−^, and CD3^−^TCRγδ^−^ cells were identified, respectively (**G**). The CD3^−^TCRγδ^−^ non-T cell population contained CD3^−^CD8α^+^ NK cells ((**E**,**F**)). CD3^+^TCRγδ^−^ T cells contained CD4^+^ (TCRγδ^−^CD3^+^CD4^+^) and CD8α^+^ T cells (TCRγδ^−^CD3^+^CD8α^+^) (**K**). Both γδ T cells and CD8α^+^ T cells (TCRγδ^−^CD3^+^) could be further subdivided into CD8αα and CD8αβ subsets ((**D**,**I**)). The CD25 expression on γδ T cells (**H**), CD4^+^ (**L**), and CD8α^+^ T cells (**J**) were analyzed, respectively.

Based on the availability of antibodies against surface markers of chicken immune cells commercially and optimal combination of different fluorochrome conjugates (Table 1), a multi-parameter flow cytometry was established, allowing us to simultaneously define different T cell subsets, NK cells, and their activation state or antigen-presenting cells (APCs) including B cells, monocytes/macrophages in one panel. All the antibodies used were titrated to an optimal concentration. A gating strategy for immunophenotyping antigen-presenting cells and T cell subsets are shown in Figure 1 and Figure 2, respectively. For Panel 1, CD45-positive leukocytes were gated (Figure 1A) and singlet cells were chosen (Figure 1B), then dead cells were excluded (Figure 1C). Next, by outputting forward scatter (FSC) versus side scatter (SSC), lymphocytes were located in a dot-plot (Figure 1F) and Bu-1^+^ B cells were defined (Figure 1I). By excluding B cells (Figure 1E) and outputting KUL01 vs MHC-II, we could define KUL01^+^ cells (Figure 1D), which were MHC-II-positive, and consisted of two subsets, KUL01^lo^MHC-II^hi^ and KUL01^hi^MHC-II^lo^ (Figure 1G), and further analysis showed that KUL01^lo^MHC-II^hi^ subsets, but not KUL01^hi^MHC-II^lo^, partially expressed CD11c (Figure 1H).

For panel 2, as shown in Figure 2, lymphocytes were located by FSC/SSC in a dot-plot (Figure 2A), and then the output CD3 vs TCRγδ, CD3^+^TCRγδ^+^ T cells (γδ T), TCRγδ^−^CD3^+^ T cells, and CD3^−^TCRγδ^−^ non-T cells were defined, respectively (Figure 2D). The CD3^−^TCRγδ^−^ non-T cells population contained CD3^−^CD8α^+^ natural killer (NK) cells that do not express CD4 (Figure 2F,E) as previously reported [34]. γδ T cells (CD3^+^TCRγδ^+^) included CD8αβ^+^ and CD8αα^+^ subsets (Figure 2D) and expressed CD25 (Figure 2H). CD3^+^TCRγδ^−^ T cells comprised CD4^+^ and CD8α^+^ T cells (Figure 2K). CD8α^+^ T cells (CD3^+^TCRγδ^−^) could also be subdivided into CD8αβ^+^ and CD8αα^+^ subsets (Figure 2I) and barely expressed CD25 (Figure 2J), whereas TCRγδ^−^CD4^+^ T cells expressed CD25 apparently (Figure 2L).

### 3.2. The Distribution of Immune Cells in Organs of Chicken at Steady State

**Figure 3:**
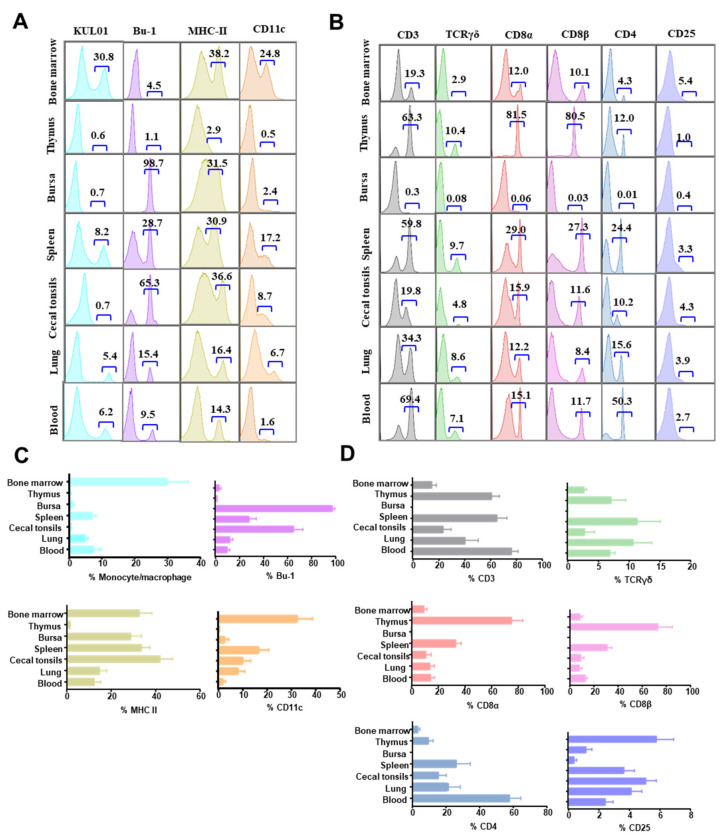
The distribution of immune cells in the organs of chicken at steady state. Mononuclear cells were isolated from different organs of 4 weeks-old naïve chickens and were surface stained with antibody Panel 1 or Panel 2. Representative staining profiles of KUL01^+^, Bu-1^+^, MHC-II^+^, and CD11c^+^ cells by tissue, are depicted as histogram plots (**A**). Representative staining profiles of CD3^+^, TCRγδ^+^, CD8α^+^, CD8β^+^, CD4^+,^ and CD25^+^ cells by tissue, depicted as histogram plots (**B**). Results for bone marrow, thymus, bursa, spleen, cecal tonsils, lung, and blood are shown. The percentages of B lymphocytes and T cell subsets are derived from a lymphocyte gate based on FSC versus SSC. Percentages of KUL01^+^, MHC-II^+^, putative CD11c^+^ cells from a leukocyte gate based on CD45. Bar chart (mean ± SD) represents total frequency of positive events for each surface marker ((**C**,**D**)) (*n* = 6).

As the relative expression of each marker in a given organ/tissue of chicken has not been detected simultaneously, we first measured the leukocyte composition within lymphoid and non-lymphoid tissues in naïve birds (steady state) using the established multicolor flow cytometry. The overall expressions and frequencies of KUL01^+^, Bu-1^+^, MHC-II^+^, putative CD11c^+^, CD3^+^, CD8α^+^, CD8β^+^, TCRγδ^+^, CD4^+^, and CD25^+^ cells in different organs/tissues are shown in Figure 3. Intriguingly, the frequencies of cells expressing specific markers varied dramatically in different organs. The KUL01^+^, MHC-II^+,^ and CD11c^+^ cells were simultaneously detected in bone marrow, spleen, and lung but were most enriched in bone marrow (30.8%, 38.2%, and 24.8%, respectively) (Figure 3A) and less dominant in the spleen (8.2%, 30.9%, and 17.2%, respectively), and lung (5.4%, 16.4%, and 6.7%, respectively). Although KUL01^+^ and MHC-II^+^ cells were detected in the blood, there were very few CD11c^+^ cells. Bu-1^+^ B cells are enriched in the bursa (98.7%) and cecal tonsils (65.3%) and account for around 31.5% MHC-II^+^ cells in the bursa and showed a hierarchy of percentage in the spleen (28.7%), lung (15.4%), blood (9.5%), bone marrow (4.5%), and thymus (1.1%) (Figure 3A,C). There were very few CD11c^+^ cells in the bursa (2.4%) but a relatively higher percentage of putative CD11c^+^ cells in cecal tonsils (8.7%). As anticipated, there were very few B cells and myeloid cells (KUL01^+^, MHC-II^+^, and CD11c^+^) in the thymus-the organ of T cell development (Figure 3A,C).

In contrast, CD3^+^ total T cells were most dominant in the thymus (63.3%), spleen (59.8%), and blood (69.4%), with a lower percentage in bone marrow (19.3%), cecal tonsil (19.8%), and lung (34.3%), respectively (Figure 3B,D). However, the proportion of T cell subsets (CD8α^+^, CD8β^+^, and CD4^+^) varied in different organs. While the ratio of CD8 to CD4 were between the normal range of 0.8–1.5 in spleen (29%/24.4%), cecal tonsil (15.9%/10.2%), and lung (12.2%/15.6%), there was a very low CD8/CD4 ratio (15.1%/50.3%) in the blood because of the high percentage of CD4 T cells and considerably high CD8/CD4 ratio (81.5%/12%) in the thymus because of the unusually low percentage of CD4 T cells (Figure 3B). The percentage of CD8α^+^ T cells was higher than that of CD8β^+^ T cells in most of organs tested, suggesting a proportion of CD8 T cells may use the CD8αα co-receptor (CD8β^−^) (Figure 3B). The frequency of γδ T cells ranged from 2.9% to 10.4% with a hierarchy of thymus > spleen > lung > blood > cecal tonsil > bone marrow. As in the organ of B cell development in birds, bursa had barely any T cells. There was a baseline expression of CD25 in these organs except in the thymus and bursa (Figure 3B).

Based on the gating strategy in Figure 2, we further examined the distributions of CD3^−^CD8α^+^ NK cells, CD8αα^+^, and CD8αβ^+^ γδ T cells, CD8αα^+^ and CD8αβ^+^ T cells, and CD4^+^CD25^+^ regulatory T cells in different organs (Table 2). There were very few NK cells (less than 1%) in tested organs, with a relatively high abundance in cecal tonsil (1%) and lung (0.8%), and no NK cells detectable in the thymus. The distribution of CD8αα^+^ and CD8αβ^+^ γδ T cells resembled that of total γδ T cells, with the highest percentage in the spleen and lung. While CD8αβ^+^ T cells were distributed in a hierarchy of thymus > spleen > blood > cecal tonsil > bone marrow > lung, CD8αα^+^ T cells showed a hierarchy of thymus > spleen > cecal tonsil > lung > blood > bone marrow (Table 2). CD4^+^CD25^+^ T cells were most abundant in bone marrow, cecal tonsil, spleen, and lung and less dominant in the thymus and blood (Table 2). Collectively, these data reveal that distinct immune cells are distributed in different organs of chicken with different abundance at steady state.

### 3.3. The Dynamic Changes of Chicken Innate Immune Cells after H7N9 Infection

**Figure 4:**
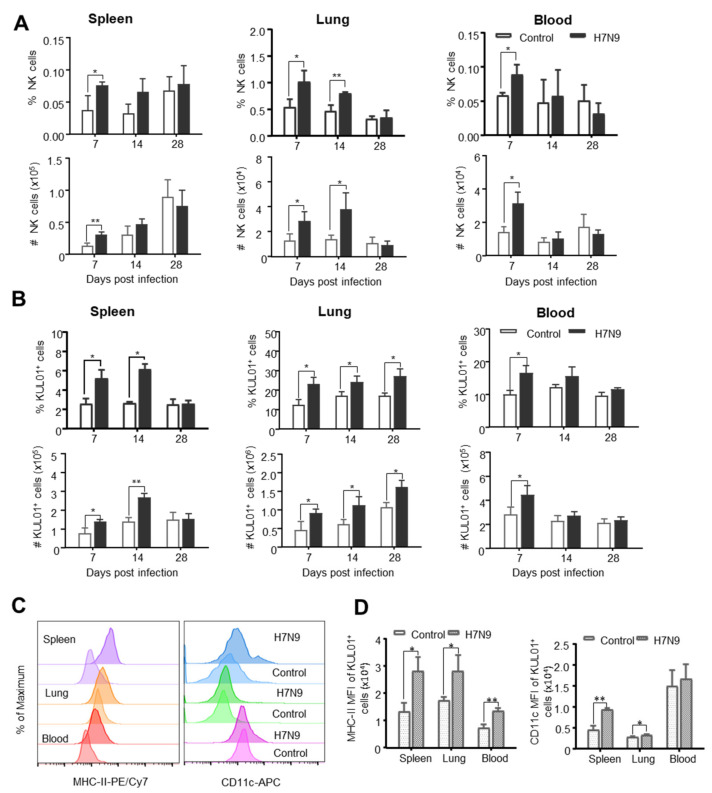
The dynamic changes of the chicken innate immune cells after H7N9 infection. Single cells were isolated from the spleen, lung, and blood of non-infected (Control) and H7N9-infected (H7N9) chickens at 7, 14, and 28 dpi and stained with antibodies from Panel 1 or Panel 2. Each subset of immune cells was analyzed by flow cytometry. The percentages and total numbers of CD3^−^CD8α^+^NK cells (**A**) and KUL01^+^ cells (**B**) in the spleen, lung, and blood of the two groups were compared, and the total number of indicated cells per organ or 5 mL blood was calculated. (**C**) Representative histogram of MHC-II and CD11c expression on KUL01^+^ cells isolated from spleens, lung, and blood of non-infected (Control) and H7N9-infected (H7N9) chickens at 14 dpi. (**D**) The bar graph shows the geometric mean of fluorescence intensity (MFI) of MHC-II and CD11c expression within KUL01^+^ cells from non-infected and H7N9 infected chickens. The values are representative of six different birds for each group. The mean ± SD value are shown (* *p* < 0.05, ** *p* < 0.01).

Since HI/VN-antibody-independent immune protection was frequently observed in the development of a vaccine against the H7N9 subtype [9,10], we are particularly interested in the changes of the cellular immune response induced by the AIV H7N9 subtype. Firstly, we examined the changes in innate immune cells in the spleen, lung, and blood at 7, 14, and 28 dpi using the established flow cytometry. As shown in Figure 4A, the percentages and absolute numbers of CD3^−^CD8α^+^NK cells significantly increased in the lung at 7 and 14 dpi and in the spleen and blood at 7 dpi, compared to the control. Similarly, the percentages and numbers of KUL01^+^ monocytes/macrophages markedly increased in the lung at 7, 14, and 28 dpi and in the spleen at 7 and 14 dpi, and in the blood at 7 dpi, compared to the control (Figure 4B). Among KUL01^+^ cells, there was a subset of KUL01^+^ cells upregulated MHC-II and CD11c expression in the spleen and lung from H7N9-infected chicken in terms of the percentage and mean fluorescence intensity (MFI) (Figure 4C,D), resembling the maturation of monocyte-derived dendritic cells observed in murine infection model [35]. These results suggested that KUL01^+^MHC-II^+^CD11c^+^ cells are potentially a subset of antigen-presenting cells in chicken after H7N9 infection. Overall, these data indicated that KUL01^+^ monocytes/macrophages, putative DC, and CD3^−^CD8α^+^NK participate in host defense against H7N9 infection.

### 3.4. The Dynamic Changes of Chicken Adaptive Immune Cells after H7N9 Infection

**Figure 5:**
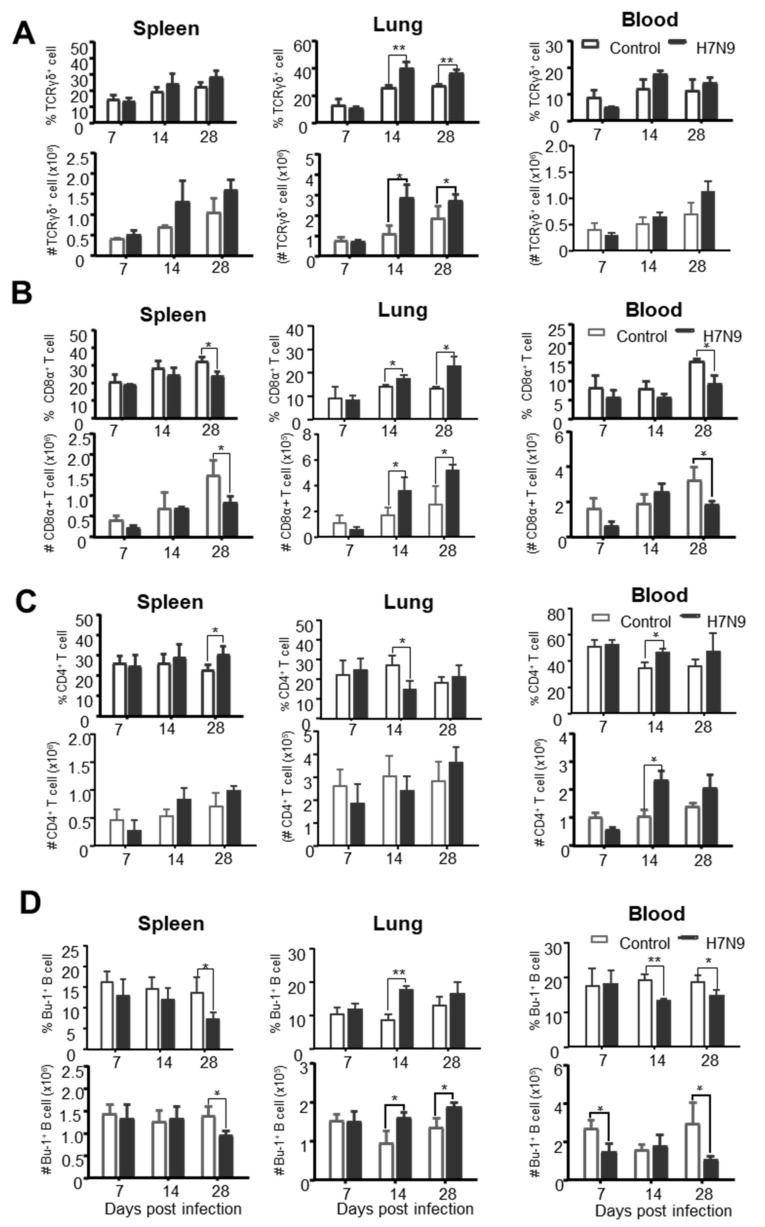
The dynamic changes of chicken’s adaptive immune cells after H7N9 infection. Single cells were isolated from the spleen, lung, and blood of non-infected (Control) and H7N9-infected (H7N9) chickens at 7, 14, and 28 dpi and stained with antibodies from panel 1 or panel 2. Each subset of immune cells was analyzed by flow cytometry. The percentages and total numbers of γδ T cells (**A**), CD8α^+^ T cells (**B**), CD4^+^ T cells (**C**), and Bu-1^+^ B cells (**D**) in spleen, lung, and blood were compared between the two groups. The total number of indicated cells per organ or 5 mL blood was calculated. The values were representative of six different birds for each group. The mean ± SD value are shown (* *p* < 0.05, ** *p* < 0.01).

Further analysis of the changes of adaptive immune cells after H7N9 infection showed that γδ T cells were significantly increased *(p* < 0.05) in the lung at 14 and 28 dpi but not in the spleen and blood at any time-point after H7N9 infection (Figure 5A). Similarly, CD8α^+^ T cells were markedly increased *(p* < 0.05) in the lung at 14 and 28 dpi but started to decrease at 28 dpi in the spleen and blood in terms of the percentage and number (Figure 5B). These results suggested that H7N9 induced a local immune response for γδ T and CD8α^+^ T cells. Differently, CD4^+^ T cells significantly increased in the blood at 14 dpi, but not much change in the spleen and lungs in terms of the numbers after H7N9 infection (Figure 5C). In contrast to T cell response, Bu-1^+^ B cells significantly decreased in the spleen and blood but increased in the lung (Figure 5D), indicating that B cells may be recruited from periphery to lung. These data suggest that γδ T cells and CD8α^+^ T cells participated in the local immune response in the lung, whereas CD4 T cell and B cell may be responsible for a systemic immune response following H7N9 infection.

### 3.5. CD25 Expression on T-Cell Subsets after H7N9 Infection

**Figure 6:**
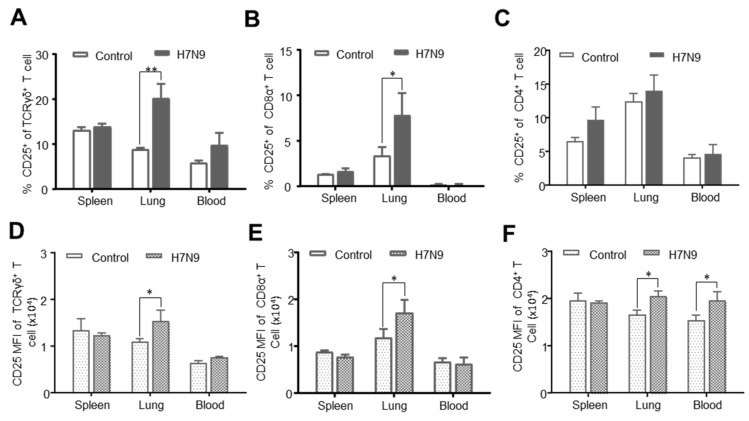
CD25 expression of chicken T cell subtypes after H7N9 infection. Single cells were isolated from the spleen, lung, and blood of non-infected (Control) and H7N9-infected (H7N9) chickens at 14 dpi and stained with antibodies from panel 2. CD25 expression on T-cell subsets was analyzed by flow cytometry. The percentages of TCRγδ^+^CD25^+^ T cells (**A**), CD8^+^CD25^+^ T cells (**B**), and CD4^+^CD25^+^ T cells (**C**) in the spleen, lung, and blood were compared between the two groups. The mean fluorescence intensity (MFI) of CD25 expression on single γδ T cells (**D**), CD8^+^ T cells (**E**), and CD4^+^ T cells (**F**) from non-infected and H7N9 infected chickens were compared. The data are representative of six different infected and non-infected birds. The mean ± SD value are shown (* *p* < 0.05, ** *p* < 0.01).

CD25 molecule is not only a marker for the identification of chicken Tregs but also for T cell activation [36]. In order to investigate the potential H7N9-induced activation of T-cell subsets, we analyzed the CD25 expression on γδ T cells, CD8α^+^ T cells, and CD4^+^ T cells. As shown in Figure 6, γδ T and CD8α^+^ T cells from H7N9-infected chicken were activated with upregulated expression of CD25 in the lung but not in the spleen and blood, compared to that of naïve birds (Figure 6A,B). The increase of MFI of CD25 on these two subsets indicated the upregulated expression of CD25 at the single-cell level (Figure 6D,E). In contrast, there was no difference in the percentages of CD4^+^CD25^+^ T cells between the two groups (Figure 6C). However, the MFI of CD25 on CD4^+^CD25^+^ T cells was significantly augmented (*p* < 0.05) in lung and blood in H7N9-infected birds (Figure 6F). These results suggested that H7N9 infection induced the activation of γδ T and CD8α^+^ T cells.

## 4. Discussion

Immune protection against AIV is mainly mediated by humoral and cellular immune responses [15]. Although HI and NA play a paramount role as a correlate of protection for conventional influenza vaccines, the efficacy of an influenza vaccine is not always correlated with the level of the humoral immune response [5,6,37]. Thus, it is necessary to define cellular correlates of immune protection for specific subtypes of AIV. In the present study, the development of a multi-parameter flow cytometry suitable for chicken/birds allowed us to comprehensively analyze the phenotypical changes of innate and adaptive immune cells in chicken, making an important advance toward identifying novel correlates of protection after immunization with vaccines against influenza and other avian viral diseases. Employing this assay, for the first time, our study revealed that γδ T and CD8 T cells are activated and, along with KUL01^+^ cells and NK cells, take part in local and systemic cellular immune responses in chicken after AIV H7N9 infection.

Polychromatic flow cytometry is a powerful tool that can be used to analyze multiple functions and phenotypes of a single cell. In chickens, although conventional immune cells such as γδ T cells, CD4, CD8 T cells, and monocytes/macrophages were defined using 2 to 4 color flow cytometry in previous studies [26,27,28], to establish a multi-parameter flow cytometry more than five colors was still challenging due to the limited availability of antibodies to chicken immune cell markers. The current combination of antibodies (6 colors for Panel 1 and 7 colors for Panel 2) enabled us to immunophenotype almost all the known, characterized distinct immune cells in chickens simultaneously. We attempted to include chicken CD44 as an antigen-experienced T cell marker as per previous studies in chicken [28] and in mammals [38], but T cells isolated from both naïve and infected birds expressed CD44^hi^ completely. Thus, CD44 cannot be used to differentiate T cell activation in outbred chicken in our experience. We also attempted to detect IFN-γ expression by T cells through intracellular cytokine staining (ICS), as previously reported [26,39,40], but we could not reproduce reliable intracellular staining of IFN-γ even though more than seven clones of anti-chicken IFN-γ antibodies that are commercially available were tested (data not shown). Further work is needed to establish a better reproducible ICS assay, as well as a cytotoxicity assay of T lymphocytes (CTL) [41] to delineate T cell function in chickens after H7N9 infections.

Chickens have a unique immune system distinct from mammals, characterized by a lack of lymph nodes, an extraordinarily high proportion of γδ T cells, and a unique organ for B cell development [42,43]. Without a lymphatic draining system, DCs may activate T cells locally without the need to migrate to lymph nodes for T cell priming that is required in mammals. Indeed, at steady state, we observed that a lot of potential DCs (KUL01^+^, MHC-II^+,^ and CD11c^+^) reside in the lung (Figure 3A,C), which could be associated with the local response of γδ T cell and CD8α^+^ T cells in the lung after infection (Figure 5). Although the literature states that the frequency of γδ T cells are extremely high in chicken [12,42,43], we only observed an average percentage of 3–10% in all the tested organs from 4-week old chicken. Surprisingly, the percentage of CD4 was so high in blood and so low in the thymus (Figure 3B,D) that it is unclear if this phenotype is a universal characteristic for chicken or not.

While the roles of each subset of immune cells were well defined in mammals during IAV infection [13,14,15], it was unclear how the chicken immune cells respond to AIV infection or immunization. The present study revealed that KUL01^+^ cells, a subset of putative DC cells, NK cells, γδ T cell, CD8α^+^ T cells, CD4 T cells, and B cells all differentially participated in the host defense against H7N9 infection. KUL01^+^ cells were thought to be mainly monocytes and/or macrophages [44]. However, a recent study clearly showed that in vitro differentiated DCs from chicken bone marrow expressed KUL01 [45], suggesting that KUL01 is not exclusively expressed on macrophages, but also on DCs. Interestingly, in this study, in combination with a putative CD11c staining, we identified a subset of KUL01^+^ cells with upregulated CD11c and MHC-II expression (KUL01^+^, MHC-II^+^, and CD11c^+^) after infection, indicating that this population might be potentially a subset of dendritic cells (Figure 4C). However, further characterization of this subset is needed in the future. In addition, a subset of conventional DCs in chicken has been identified, which was phenotypically KUL01^−^MHCII^+^CD11c^+^ and exhibited DC-specific core gene expression signatures such as XCR1, ZBTB46, Flt3, and so on [46]. Of note, the anti-CD11c monoclonal antibody (clone 8F2) used in that study is not commercially available and different from the one we used in the present study. The identity of the DC subset identified in this study would be warranted by an in-depth analysis of DC-specific core gene expression signatures. Yu et al. found that splenic KUL01^+^MHC-II^+^ cells may act as M1 and M2-like macrophages in chickens [47], however, in the present study, this population was identified only in 1 out of 3 chickens and not observed in the lung. Chicken NK cells were mainly defined as CD3^−^CD8α^+^ [24,48,49]. A previous study showed that and LPAIV H9N2, but no HPAIV H5N1 was able to induce the increase and activation of CD3^−^CD8α^+^ NK cells in the lung [24]. As HPAIV H7N9s also emerged, it would be interesting to compare if LPAIV and HPAIV H7N9 virus activate NK cells differentially in the future. It was shown that chicken CD8 T cells mediated immune protection against H5N1 subtype [23]. In this study, chicken CD8 T cells increased and were activated after H7N9 infection; it is thus plausible to speculate that this population participates in protective immunity against H7N9 infection. Similarly, chicken γδ T cells may also play a protective role, though its role remains to be addressed after H7N9 as evidence showed that γδ T cells are able to provide heterotypic immune protection against HPAIV H5N1 infection in mice [21]. It is noteworthy that AIV H7N9 infection induced a systemic response for KUL01^+^ cells and NK cells as these cells increased in spleen, lung, and blood whereas γδ T cells and CD8α^+^ T cells responded and are activated only in the lung, suggesting AIV H7N9 infection induced a local T cell mucosal immunity. Our results also highlighted the importance to comprehensively dissect the immune response in local tissues of chicken besides PBMCs after infection [28,50].

Overall, the present study established a polychromatic flow cytometry for in-depth analysis of the changes of distinct immune cells in chickens before and after H7N9 infection. Our results for the first time disclosed that H7N9 LPAIV induces distinct local and systemic cellular immune responses in chicken following inoculation via the natural intranasal route and increased our understanding of cellular immunity of chicken to H7N9 AIV infection. The flow cytometric assay developed in this study is valuable for analyzing cellular immune responses to AIVs and other avian infectious diseases in order to establish protection criteria.

## Figures and Tables

**Table 1:** Antibodies used for flow cytometry in this study.

Marker	Clone	Isotype	Conjugate	Purpose
Panel 1				
CD45	LT40	mouse IgM	PerCP-Cy5.5	Leukocytes
Bu-1	AV20	mouse IgG1	FITC	B cells
Monocyte/macrophage	KUL01	mouse IgG1	PE	Monocytes and macrophages
MHC Class II	2G11	mouse IgG1	PE-Cy7	DC lineage marker
CD11c	polyclonal	rabbit IgG	APC	DC lineage marker
Dead Cell Stain	-	-	FVD eFluor 780	Live cell
Panel 2				
CD3	CT-3	mouse IgG1	PerCP-Cy5.5	T cells
TCRγδ	TCR-1	mouse IgG1	Biotin	γδ T cells
CD8α	CT-8	mouse IgG1	AF700	CD8^+^ T cells
CD8β	EP42	mouse IgG2a	PE	CD8^+^ T cells
CD4	CT-4	mouse IgG1	Pacific blue	CD4^+^ T cells
CD25	AV142	mouse IgG1	FITC	Activation
Streptavidin	-	-	BV510	-
Dead Cell Stain	-	-	FVD eFluor 780	Live cell

**Table 2:** Distribution of NK and T cell subsets in different organs.

Leucocyte Type	Percentage (%)						
	Bone Marrow	Thymus	Bursa	Spleen	Cecal Tonsil	Lung	Blood
CD3^−^CD8α^+^NK cells ^a^	0.5 ± 0.2 ^c^	ND ^d^	<0.1	0.3 ± 0.2	1.0 ± 1.1	0.8 ± 0.4	0.2 ± 0.1
TCRγδ^+^ T cells ^a^	5.4 ± 2.0	6.9 ± 2.3	0.12 ± 0.03	12.2 ± 3.9	4.8 ± 1.9	13.6 ± 3.2	6.3 ± 2.3
TCRγδ^+^CD8αβ^+^T cells ^a^	0.8 ± 0.1	0.7 ± 0.3	<0.1	3.3 ± 2.2	0.4 ± 0.3	2.4 ± 1.3	0.5 ± 0.4
TCRγδ^+^CD8αα^+^T cells ^a^	1.1 ± 0.3	<0.1	<0.1	3.8 ± 1.6	1.1 ± 0.8	2.9 ± 1.1	0.6 ± 0.3
TCRγδ^−^CD3^+^CD8αβ^+^T cells ^a^	7.4 ± 1.6	43.6 ± 7.7	<0.1	23.4 ± 8.6	8.4 ± 3.8	5.5 ± 1.9	11.7 ± 4.7
TCRγδ^−^CD3^+^CD8αα^+^T cells ^a^	0.9 ± 0.5	13.1± 9.9	<0.1	5.7 ± 2.7	2.4 ± 0.9	2.2 ± 0.9	0.8 ± 0.3
TCRγδ^−^CD3^+^CD4^+^T cells ^a^	2.7 ± 1.1	5.9 ± 2.8	<0.1	26.3 ± 7.8	10.4 ± 8.2	20.5 ± 7.2	43.8 ± 6.8
TCRγδ^−^CD4^+^CD25^+^T cells ^b^	9.8 ± 4.6	4.9 ± 2.7	ND ^d^	6.5 ± 3.5	8.5 ± 5.9	9.4 ± 2.9	1.8 ± 1.3

^a^ Percentage of T lymphocytes were derived from a lymphocyte gate based on forward scatter (FSC) versus side scatter (SSC). ^b^ Percentages of CD4^+^CD25^+^ T cells are expressed as a percentage out of CD4^+^ T cells. ^c^ Values are means ± SD. *n* = 6. ^d^ ND, not detected.
